# Staphylococcus aureus With Reduced Vancomycin Susceptibility: Diagnostic Challenges in Clinical Settings

**DOI:** 10.7759/cureus.108661

**Published:** 2026-05-11

**Authors:** Mithali B Jadhav, Geeta Karande, Satish Patil

**Affiliations:** 1 Department of Microbiology, Krishna Institute of Medical Sciences, Krishna Vishwa Vidyapeeth (Deemed to Be University), Karad, IND

**Keywords:** antibiotic resistance, antimicrobial susceptibility testing, clinical microbiology, diagnostic challenges, staphylococcus aureus, vana operon, vancomycin-intermediate staphylococcus aureus (visa), vancomycin-non-susceptible s. aureus, vancomycin resistance mechanisms, vancomycin-resistant staphylococcus aureus (vrsa)

## Abstract

Vancomycin-intermediate *Staphylococcus aureus *(VISA) and vancomycin-resistant *S. aureus* (VRSA) have become significant challenges in treating staphylococcal infections, especially in areas where methicillin-resistant *S. aureus* (MRSA) is highly prevalent. Although vancomycin has long been a crucial treatment for serious MRSA infections, strains exhibiting reduced susceptibility or complete resistance have emerged due to its extensive use. While VISA arises through a progressive adaptive mechanism marked by cell wall thickening, altered autolytic activity, and mutations in various stress-response regulatory pathways, VRSA typically develops through horizontal transfer of the vanA operon, most frequently from vancomycin-resistant enterococci (VRE). These changes make it harder for vancomycin to reach its intended locations, which could lead to therapy failure and insufficient bacterial eradication. Prolonged use of vancomycin, the existence of chronic ulcers, the use of invasive medical devices, underlying renal illness, and concurrent colonization with MRSA and VRE are factors that contribute to the development of VISA and VRSA.

The true incidence of VISA and VRSA is still unclear, despite their clinical significance. Routine susceptibility testing inadequacies, vancomycin's limited agar diffusion, borderline minimum inhibitory concentration values, and the inconsistent dependability of automated systems all contribute to ongoing diagnostic challenges. Underdiagnosis is further brought about by inadequate screening programs. For the purpose of guiding appropriate medication, preventing transmission, and assisting with efficient infection control measures, timely and accurate detection is essential. To address the growing threat of VISA and VRSA, there is an urgent need for continuous surveillance, improved diagnostic techniques, and the development of novel antimicrobial agents, as the availability of effective treatment options continues to decline.

## Introduction and background

Severe *Staphylococcus aureus* infections remain a serious clinical problem worldwide, affecting patients in both community settings and healthcare facilities [1]. After β-lactam antibiotics were introduced, *S. aureus*, once thought to be easily treatable, rapidly developed resistance to penicillin, drastically altering treatment approaches over the previous 50 years [1,2]. As viable treatment choices continue to diminish, the emergence and spread of methicillin-resistant *Staphylococcus aureus* (MRSA) have increased these concerns [2].

The majority of clinical *S. aureus* isolates in many regions exhibit penicillin resistance, and a major fraction also exhibit methicillin resistance [2]. MRSA accounts for approximately 25%-50% of *S. aureus* infections in hospital settings across various regions, reflecting its substantial epidemiological burden. The emergence of community-associated MRSA strains, some of which produce potent toxins and possess unique genetic characteristics that can cause serious infections even in apparently healthy individuals, has further increased the global impact of MRSA [2,3].

Vancomycin, a glycopeptide antibiotic, was the cornerstone of treatment for serious MRSA infections for many years. However, isolates with reduced susceptibility have emerged due to long-term reliance on vancomycin and its extensive use in clinical settings. The discovery of the first clinical isolate of vancomycin-intermediate *S. aureus* (VISA) in 1997 marked a dramatic shift in MRSA epidemiology [3].

Once thought to be rare, VISA strains are now being detected more frequently in a number of nations, raising concerns about their growing prevalence. Subsequent results from several monitoring studies have shown VISA rates higher than initially anticipated, suggesting that intermediate-resistance phenotypes may be more common than previously estimated [4].

The emergence of vancomycin-resistant *Staphylococcus aureus* (VRSA), which shows total resistance to vancomycin and is mostly caused by acquisition of the vanA operon from vancomycin-resistant *enterococci* (VRE), is even more worrisome. Although there are still a few documented cases of VRSA, their clinical implications are substantial. Patients with VRSA infections frequently have chronic wounds, underlying comorbidities, and a history of long-term antibiotic use, all of which promote bacterial survival and foster environments that are favorable to horizontal gene transfer [4,5].

This narrative review will highlight the emergence of VISA and VRSA as major concerns to clinical management after summarizing the current understanding of MRSA prevalence in both healthcare and community settings. Particular attention will be paid to the mechanisms underlying vancomycin nonsusceptibility, the challenges of laboratory detection, the limitations of both automated and traditional diagnostic techniques, and the consequences of inadequate screening procedures. The potential for overlooked or undetected cases among colonized or high-risk individuals will also be investigated, highlighting the significance of improved surveillance and greater diagnostic precision in clinical microbiology labs [5].

The following abbreviations and their meanings are used throughout this review: 1) MRSA: strains resistant to methicillin and other β-lactam antibiotics; 2) VISA: strains showing intermediate susceptibility to vancomycin; 3) heterogeneous vancomycin-intermediate *Staphylococcus aureus* (hVISA): strains with subpopulations exhibiting intermediate vancomycin resistance; 4) VRSA: strains fully resistant to vancomycin; 5) minimum inhibitory concentration (MIC): lowest concentration of an antimicrobial that inhibits visible bacterial growth; and 6) Clinical and Laboratory Standards Institute (CLSI).

## Review

VISA/VRSA: a diagnostic difficulty

In clinical microbiology, vancomycin-nonsusceptible *Staphylococcus aureus* (VISA and VRSA) is increasingly recognized as a significant diagnostic challenge. Recent research indicates that MRSA variants with reduced susceptibility to vancomycin are emerging in both hospital and community settings, despite MRSA traditionally being associated with healthcare-related infections [6,7]. These strains are frequently identified in patients with invasive devices, chronic wounds, and prolonged antibiotic exposure, but they have also been reported in community-associated infections [7,8]. VISA isolates have demonstrated MIC values close to clinical breakpoints in several studies. However, conventional laboratory methods often fail to detect them accurately, resulting in diagnostic and therapeutic challenges [8,9].

Methods

Study Design

This review is organized as a narrative analysis that emphasizes the epidemiological backdrop, laboratory identification, and diagnostic obstacles related to vancomycin-nonsusceptible *Staphylococcus aureus* (VISA/VRSA).

Eligibility Criteria

Inclusion criteria: Studies, case reports, and clinical guidelines that address the rise, laboratory identification, and clinical ramifications of VISA, VRSA, and hVISA.

Exclusion criteria: Research that is not pertinent to vancomycin susceptibility, outdated diagnostic methods, or those that do not concentrate on *Staphylococcus aureus*.

Information Sources

A literature search was performed using PubMed/MEDLINE and Google Scholar. Google Search was employed to gather epidemiological research and clinical guidelines (e.g., CLSI).

Search Strategy

The investigation included terms such as "MRSA", "VRSA", "VISA", "hVISA", and "MIC". Additionally, the search encompassed laboratory techniques for both phenotypic and molecular identification.

Study Selection Procedure

The articles were initially evaluated based on their titles and abstracts to determine their relevance to *S. aureus* vancomycin susceptibility and diagnostic testing. Subsequently, full-text articles were reviewed to confirm eligibility, with a particular focus on studies addressing diagnostic challenges and resistance mechanisms.

Quality Assessment/Risk of Bias

The quality of the studies was assessed based on their design and methodology. To reduce bias, priority was given to peer-reviewed research and official guidelines over unverified reports.

Data Extraction Method

Key information, including publication details, study locations, specific diagnostic techniques (such as broth microdilution and automated systems), and MIC values, was manually gathered from the selected full-text articles.

Data Synthesis Approach

A narrative synthesis was employed to thematically organize the gathered findings, focusing on resistance mechanisms, diagnostic limitations, and classification inconsistencies, to provide a clear and comprehensive summary.

Vancomycin susceptibility ranges and problems with classification

The majority of *S. aureus* isolates were at doses between 0.5 and 2 μg/mL. Current CLSI guidelines classify *S. aureus* isolates as vancomycin-resistant (VRSA) if their MIC values are 16 μg/mL or higher, and vancomycin-intermediate (VISA) if their MIC values are 4-8 μg/mL [10,11]. However, over time, different regulatory bodies have adopted varying classification limitations and interpretation standards [10-12]. Isolates with an MIC of 4 μg/mL were deemed susceptible under previous CLSI standards. However, the Centers for Disease Control and Prevention has regularly recommended that these isolates be interpreted cautiously and undergo additional confirmatory testing. Clinical evidence further indicates that, especially in patients with deep-seated or endovascular infections, isolates having MIC values at the upper limit of the susceptible range (2 μg/mL) may exhibit decreased response to vancomycin therapy. Misclassification of isolates and delayed discovery of resistance in clinical practice have resulted from these discrepancies in interpretation criteria [12,13].

Emergence of heterogeneous VISA and VISA

*S. aureus* strains with modest but clinically significant increases in vancomycin MIC values have progressively surfaced over the last 20 years. hVISA is an early stage in which an apparently susceptible isolate contains tiny groups of resistant cells [14,15]. In conventional laboratory testing, these strains often appear fully susceptible, but in reality, they harbor small populations of highly resistant cells that can proliferate under selective pressure [15,16].

Early studies showed that hVISA strains can produce daughter populations that progressively acquire higher levels of resistance when exposed to increasing vancomycin concentrations [16,17]. Later-identified true VISA isolates exhibit consistently intermediate MIC values over the whole bacterial population. Patients receiving long-term vancomycin therapy for recurrent MRSA infections frequently develop these strains. According to reports from tertiary care facilities worldwide, VISA is more prevalent than previously believed, and its underdetection is largely due to reliance on automated systems and limitations in phenotypic testing [17].

The rise of VRSA

Confirmed instances of VRSA are still very uncommon but are clinically problematic, even though VISA is being identified with increasing frequency. The vanA operon, which is frequently acquired from VRE, is typically responsible for high-level resistance [18,19]. Since long-term comorbidities such as diabetes, peripheral vascular disease, chronic ulcers, and renal impairment promote bacterial persistence and interspecies contact, documented VRSA instances have primarily been recorded in individuals with these disorders [19,20]. Using broth microdilution, isolates in reported cases showed MIC values ≥32 μg/mL, indicating total resistance [20,21].

Case studies frequently revealed that individuals had several VRE infections or colonization at the same time. Genetic investigations clearly suggest that in vivo horizontal gene transfer across several bacterial species is how VRSA emerges, resulting in the development of high-level resistance. A multiresistant conjugative plasmid containing the vanA transposon was discovered to transfer from VRE to MRSA in a number of individuals, resulting in high-level vancomycin resistance. These results underscore the significance of targeted screening for co-colonization in high-risk patients [21].

Mechanisms of decreased susceptibility to vancomycin

Vancomycin and other glycopeptides act by binding to the D-Ala-D-Ala termini of peptidoglycan precursors, thereby inhibiting bacterial cell wall synthesis. Rather than resulting from a single gene, resistance in VISA arises through multiple physiological adaptations [22,23]. VISA strains produce large amounts of poorly cross-linked peptidoglycan, which prevents vancomycin from reaching its target by trapping it within the outer layers of the cell wall. Studies on VISA strains have demonstrated thickened cell walls, reduced autolytic activity, increased peptidoglycan synthesis, and structural alterations, including changes in muropeptide composition [23].

VRSA, in contrast, develops resistance by modifying the cell wall target site. Upon acquisition of the vanA gene, the terminal D-Ala-D-Ala is replaced by D-Ala-D-Lac, thereby significantly reducing vancomycin's binding affinity. This biochemical modification is associated with mobile genetic elements capable of transferring between bacterial species, leading to high-level resistance. The vanA gene is typically associated with MIC values ≥16 µg/mL. In contrast, the vanB gene cluster also leads to D-Ala-D-Lac formation but confers variable and generally lower levels of resistance and is rarely identified in *S. aureus* [24].

Vancomycin reduced susceptibility in healthy carriers

Reduced susceptibility to vancomycin may not be exclusive to clinical infections, according to new research. Coagulase-negative *staphylococci *from healthy carriers demonstrated unstable heteroresistance to vancomycin in a number of investigations, but after multiple passes, they reverted to susceptibility [25,26]. Vancomycin exposure rapidly selected for resistant subpopulations despite the instability of this resistance, demonstrating that resistance can resurface swiftly under antibiotic pressure. These results highlight the lack of comprehensive screening among high-risk groups, including residents of long-term care facilities, patients with chronic wounds, and immunocompromised people, and raise concerns about the existence of glycopeptide-intermediate *staphylococci *in the community [26].

Diagnostic methods and vancomycin susceptibility testing

Standardized, proven reference procedures are necessary for accurate detection of VISA and VRSA. The gold standard for figuring out MIC values is still broth microdilution and agar dilution. The broth microdilution method employs microtiter plates containing stepwise antibiotic concentrations to determine the MIC, defined as the lowest concentration that prevents visible bacterial growth. Based on vancomycin MIC values, *S. aureus* is classified as vancomycin-susceptible *S. aureus* at ≤2 µg/mL, VISA at 4-8 µg/mL, or VRSA at ≥16 µg/mL. Small colonies that form within the inhibition zones may affect how the E-test, which uses a standardized 0.5 McFarland inoculum, interprets gradient MIC values. Only VRSA cases with extremely high MIC values can be reliably identified by disk diffusion alone; it is not appropriate for VISA detection [27,28].

Although automated methods like VITEK (bioMérieux, Marcy-l'Étoile, France) and MicroScan (Beckman Coulter, Brea, CA) are frequently employed, their performance in identifying VISA and VRSA has been variable. Automated techniques failed to discover isolates with verified intermediate or resistant MICs in multiple studies, especially those in the 4-8 μg/mL range. To increase detection sensitivity, further screening with brain-heart infusion agar containing 6 μg/mL vancomycin is advised [27]. The presence of visible bacterial growth on this medium indicates reduced susceptibility to vancomycin and suggests possible VISA. These isolates should be further confirmed using MIC-determination methods such as broth microdilution or the E-test. Absence of growth indicates susceptibility to vancomycin. It might be required to change screening procedures and think about employing lower vancomycin concentrations on selective media due to the continuous uncertainty in interpreting borderline MIC values [27,28].

## Conclusions

The diagnostic method for identifying VISA and VRSA is intricate and is constantly changing. The underrecognition of these resistant strains is mostly due to misclassification brought on by limitations in phenotypic testing, overreliance on automated methods, and inadequate screening techniques. Conditions that promote decreased susceptibility to vancomycin are created by the increasing frequency of MRSA and selection pressure from glycopeptide use. Increased monitoring, targeted screening of high-risk individuals, and cautious interpretation of susceptibility data are crucial. To effectively treat VISA, VRSA, and other glycopeptide-resistant *staphylococci*, new antimicrobial medicines and improved diagnostic techniques are ultimately desperately needed.
